# Metastatic model of HPV+ oropharyngeal squamous cell carcinoma demonstrates heterogeneity in tumor metastasis

**DOI:** 10.18632/oncotarget.8254

**Published:** 2016-03-22

**Authors:** Daniel W. Vermeer, Joseph D. Coppock, Erliang Zeng, Kimberly M. Lee, William C. Spanos, Michael D. Onken, Ravindra Uppaluri, John H. Lee, Paola D. Vermeer

**Affiliations:** ^1^ Cancer Biology Research Center, Sanford Research, Sioux Falls, South Dakota, USA; ^2^ Department of Biology, University of South Dakota, Vermillion, South Dakota, USA; ^3^ Department of Computer Science, University of South Dakota, Vermillion, South Dakota, USA; ^4^ Department of Otolaryngology/Head and Neck Surgery, Sanford Health, Sioux Falls, South Dakota, USA; ^5^ Department of Cell Biology and Physiology, Washington University School of Medicine, St. Louis, Missouri, USA; ^6^ Otolaryngology, Washington University School of Medicine, St. Louis, Missouri, USA

**Keywords:** head and neck oral cancer, human papillomavirus, metastasis, recurrence

## Abstract

Human papillomavirus induced (HPV+) cancer incidence is rapidly rising, comprising 60–80% of oropharyngeal squamous cell carcinomas (OPSCCs); while rare, recurrent/metastatic disease accounts for nearly all related deaths. An *in vivo* pre-clinical model for these invasive cancers is necessary for testing new therapies. We characterize an immune competent recurrent/metastatic HPV+ murine model of OPSSC which consists of four lung metastatic (MLM) cell lines isolated from an animal with HPV+ OPSCC that failed cisplatin/radiation treatment. These individual metastatic clonal cell lines were tested to verify their origin (parental transgene expression and define their physiological properties: proliferation, metastatic potential, heterogeneity and sensitivity/resistance to cisplatin and radiation. All MLMs retain expression of parental HPV16 E6 and E7 and degrade P53 yet are heterogeneous from one another and from the parental cell line as defined by Illumina expression microarray. Consistent with this, reverse phase protein array defines differences in protein expression/activation between MLMs as well as the parental line. While *in vitro* growth rates of MLMs are slower than the parental line, *in vivo* growth of MLM clones is greatly enhanced. Moreover, *in vivo* resistance to standard therapies is dramatically increased in 3 of the 4 MLMs. Lymphatic and/or lung metastasis occurs 100% of the time in one MLM line. This recurrent/metastatic model of HPV+ OPSCC retains the characteristics evident in refractory human disease (heterogeneity, resistance to therapy, metastasis in lymph nodes/lungs) thus serving as an ideal translational system to test novel therapeutics. Moreover, this system may provide insights into the molecular mechanisms of metastasis.

## INTRODUCTION

Head and neck squamous cell carcinoma (HNSCC) is the sixth most common cancer worldwide and although clinical therapies have improved, local recurrence and metastasis have stagnated the overall prognosis at 50% survival for decades [1, 2]. A subset of these cancers, oropharyngeal squamous cell carcinomas (OPSCC), are increasing at near epidemic rates [3]. In the majority of these cases (60–80%) human papillomavirus (HPV) is the causative agent [4]. Despite highly successful clinical management of primary OPSCC disease (80–90% five year survival), loco-regional spread and distant metastasis remain the main cause of mortality for HPV+ OPSCC patients [5–6]. These clinical findings emphasize the need for establishing and characterizing a physiologically relevant animal model of metastatic HPV+ OPSCC. Recent studies have increased the mechanistic understanding of metastasis however, the lack of clinical survival benefit underscores that this knowledge remains incomplete. *In vivo* pathways governing the “invasion-metastasis cascade” [7] include: invasion, intravasation, survival of circulating tumor cells, extravasation, microscopic induction and subsequent macroscopic outgrowth at a secondary site. These biologically complex events are difficult to model *in vitro*; moreover, epithelial-mesenchymal plasticity is profoundly influenced by non-tumor cells including endothelial, fibroblasts, stromal, and infiltrating immune cells in the tumor microenvironment [8]. Additionally, cytokine and chemokine signals from distant organs influence tumor cell exosome secretion, thus establishing unique secondary organ niches capable of sustaining metastatic tumor growth [9–10]. Thus, in addition to yielding mechanistic insights into metastasis, disease specific animal models that faithfully replicate clinical disease progression as well as resistance to therapy, and route/site of metastatic outgrowth are essential for defining prophylactic and metastatic treatment regimens.

In this article, we characterize a new transplantable syngeneic mouse model of metastatic HPV+ OPSCC. Four unique metastatic cell lines were isolated from an HPV+ murine model previously described by our laboratory [11]. The parental mouse oropharyngeal epithelial cells stably transformed with HPV16 E6 and E7 together with hRas and luciferase, (mEERL) and the newly derived mEERL lung metastasis cell lines (MLMs) maintain the cellular effects of these driver oncogenes. Notably, the MLM cell lines possess heterotypic traits in both their physiologic and molecular characteristics, replicating the heterogeneity widely described of metastatic tumors [12–13]. Additionally, these cell lines display differences in sensitivity to standard of care treatment modalities (cisplatin and radiation) and more aggressive growth *in vivo* than their parental cells, consistent with two common characteristics of metastatic cancers [14]. Finally, when re-implanted in immune competent mice, the MLM cell lines metastasize at an increased rate developing metastatic outgrowth within a reasonable time frame (30–40 days). Importantly, MLM metastasis mimics the sites of spread occurring in human disease (draining lymph nodes and lung). Finally, not only do the parental mEERL cells share characteristics with human HPV+ OPSCCs but so do the MLM cell lines. The combination of these characteristics suggests that this unique metastasis model holds great translational potential for testing new adjuvant therapies for HPV+ OPSCC.

## RESULTS

### Isolation of tumor clones

**Figure 1:**
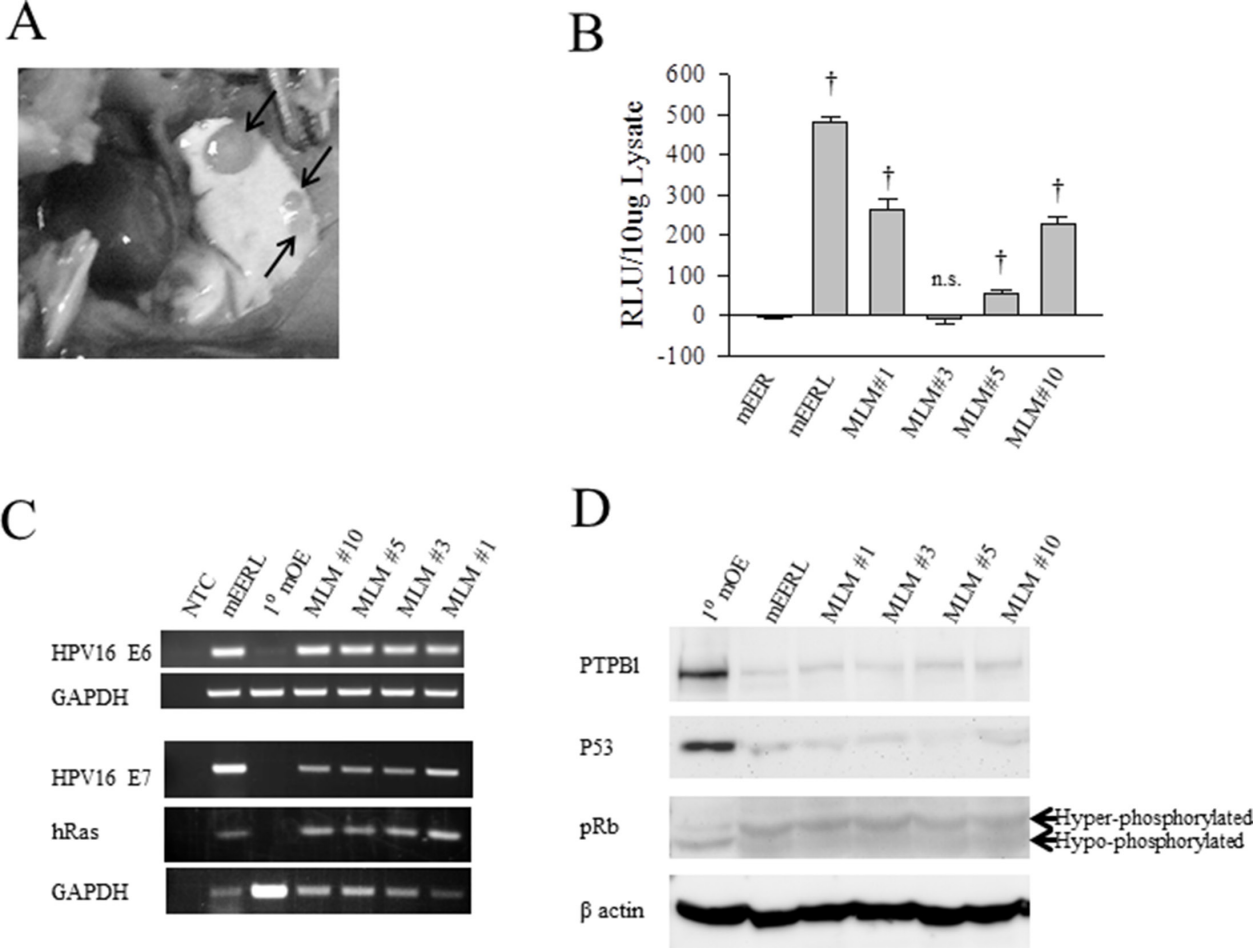
Identification of lung tumors as mEERL lung metastasis A C57 Bl/6 mouse previously injected with mEERL cells presented with ascites and was dissected on day 96 after tumor injection. (**A**) Photograph showing lung with several metastases (arrows). (**B**) Comparison of luciferase expression in mEER cells, not stably expressing luciferase, with parental mEERL line and the MLM clones. Luciferase expression measured as relative light units (RLU) per 10 ug of lysate. Each bar represents an *N* = 3; values, means ± SEM. Statistically significant differences based on ANOVA compared to mEER control: ^†^*P* ≤ 0.001. Experiments were repeated three times with similar results. (**C**) PCR analysis of HPV 16 E6, E7 and hRas expression in parental mEERL cells and primary mouse oropharyngeal epithelial cells (1°MOE) compared to the MLM clones. GAPDH was used as a loading control. (**D**) Western blot analysis of 1° mouse oropharyngeal epithelial cells (1°MOE) with parental mEERL cells and the MLM clones. The cellular effects of HPV16 E6 are shown in PTPBl and P53 expression. Cellular effects of HPV16 E7 are shown by levels of hyperphosphorylated Rb (pRb). β actin used as loading control.

During routine tumor measurements for a mouse study investigating the role of HPV16 E6/E7 in OPSCC, one animal with a late growing recurrent tumor developed ascites. This mouse had been injected with 1 × 10^6^ mEERL cells [15] and treated with cisplatin/radiation therapy (CRT): three weekly doses of cisplatin (20 mg/kg) and x-ray radiation (8 Gy) on days 10, 17, and 24. Although tumor volume measurements suggested the mouse had cleared its disease, residual tumor outgrowth became evident at day 96. Upon reaching sacrifice criteria, post mortem dissection revealed numerous lung tumors (Figure 1A). The lungs were removed and individual tumors isolated. Twelve lung tumors were harvested and tentatively named mEERL Lung Metastasis clones (MLM). Tumors were dissociated, seeded and expanded *in vitro*; five clones survived of which one, MLM#7, senesced. The four remaining clones were epithelial in morphology (Supplementary Figure 1) and further characterized.

### Identification of lung tumors as mEERL metastatic clones

To verify that the MLM clones metastasized from the originally implanted parental mEERL tumor, MLM cell lines were assayed for mEERL transgene expression. The parental mEERL cells stably express HPV16 E6/E7, hRas, and luciferase. All MLM clones, except MLM#3, harbor significant luciferase expression (*p* ≤ 0.001) (Figure 1B). mEER cells (stably expressing HPV16 E6/E7 and hRas), parent to mEERL cells, served as control. PCR for HPV16 E6, E7 and hRas confirmed their presence in all four MLM clones (#1, #3, #5 and #10) (Figure 1C). Primary mouse oropharyngeal epithelial (1°MOE) cells serve as a negative control.

The oncogenic functions of HPV16 E6 and E7 in the MLM cells were analyzed as follows. PTPBl (mouse ortholog of the human PTPN13 phosphatase) interacts with E6 resulting in phosphatase degradation [16]. All MLM cell lines demonstrate PTPBl degradation similar to the parental mEERL line. Moreover, HPV16 E6 expression correlates with loss of P53 in all the MLM clones as in the parental mEERLs. Finally, the effect of E7, hyperphosphorylation of Rb, occurs in all MLM clones (Figure 1D). As expected, none of these changes occur in the negative control, 1°MOE. Interestingly, all MLM lines showed some degree of luciferase expression silencing (MLM#3 silencing luciferase completely) while retaining HPV16 E6 and E7 function. These data suggest that luciferase expression is not necessary or required for survival of the MLM cell lines. However, the fact that they all retain expression of E6 and E7 suggests the absolute requirement of these HPV oncogenes for their survival. Taken together, the data demonstrate that the MLM clones are true metastatic cell lines derived from the parental mEERL tumor.

### Molecular profiles of the MLM clones show significant tumor heterogeneity

**Figure 2:**
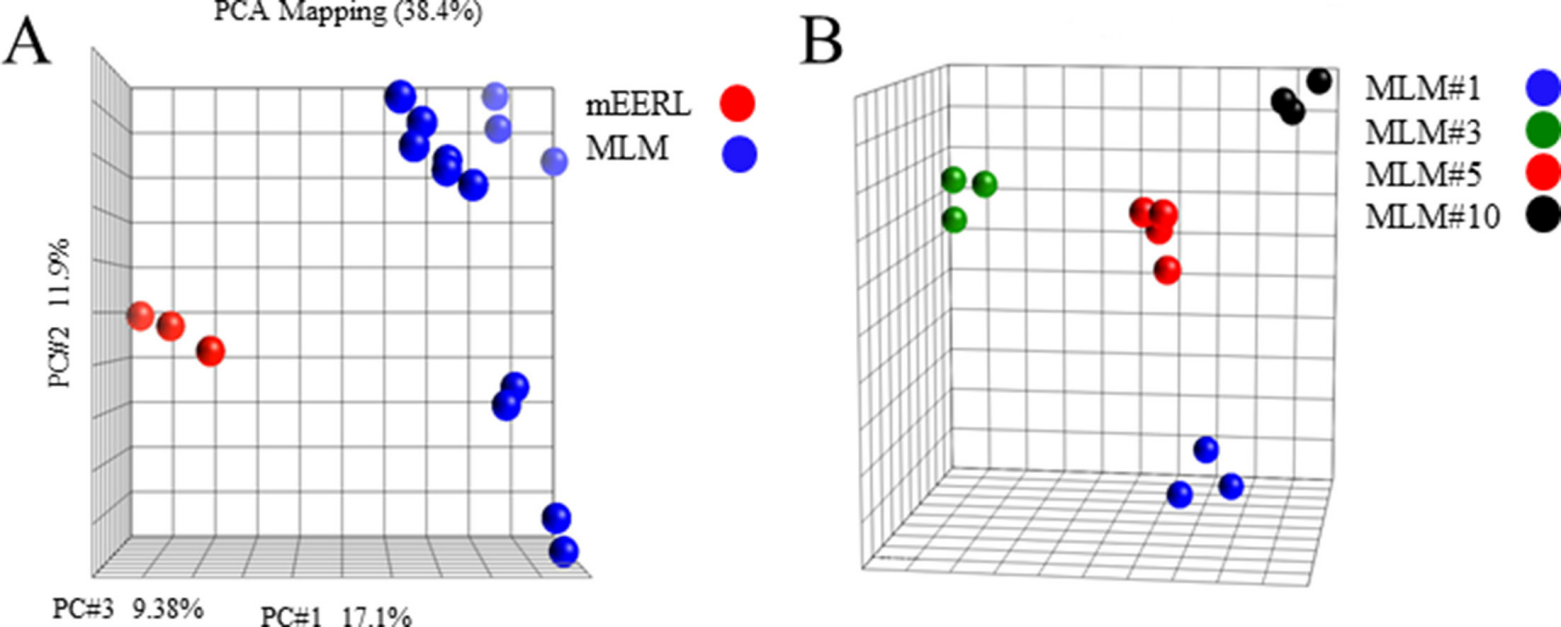
Illumina microarray (**A**) Principal component analysis of parental mEERL cells (red) compared to MLM clones (blue). (**B**) Principal component analysis of the MLM clones.

Using Illumina expression microarray analysis we asked whether the MLM lines adequately represent the heterotypic nature of metastasis [17–18]. Principal component analysis showed that the mEERL samples were very distinct from MLM lines (Figure 2A). When MLM lines were analyzed alone, significantly fewer gene expression differences were present. However upon subgroup analysis, the MLMs clustered equidistantly from each other, demonstrating genetic heterogeneity among the metastatic lines (Figure 2B).

There are 1,612 differentially expressed genes (DEGs) among MLMs (*p* ≤ 0.05, ANOVA with BY FDR), and 1,433 DEGs between the MLM and mEERL lines (*p* ≤ 0.05, *T*-test with BY FDR). Only 27 genes are shared between the two sets of DEGs. Clustering analysis of the two sets of DEGs and the shared set demonstrates that MLMs are distinct from their parental cell line (mEERL), and there are also differences between the MLMs (Supplementary Figure 2A and 2B respectively).

To discover pathways enriched in the two sets of DEGs and the shared set, Ingenuity Pathway Analysis (*IPA*^®^, QIAGEN Redwood City, www.qiagen.com/ingenuity) was performed. We found many signaling pathways enriched in the DEGs obtained from comparison between mEERL and MLM lines (Supplementary Table 1). Signaling pathways were also enriched in DEGs among MLMs (Supplementary Table 2). Interestingly, some signaling pathways such as IL-8 and Thrombin were enriched in both sets of DEGs. In addition to signaling pathways, a variety of other pathways are enriched in DEGs among MLMs, suggesting molecular mechanisms of heterogeneity. Finally, a collection of degradation pathways were enriched in the 27 shared genes (Supplementary Table 3).

**Figure 3:**
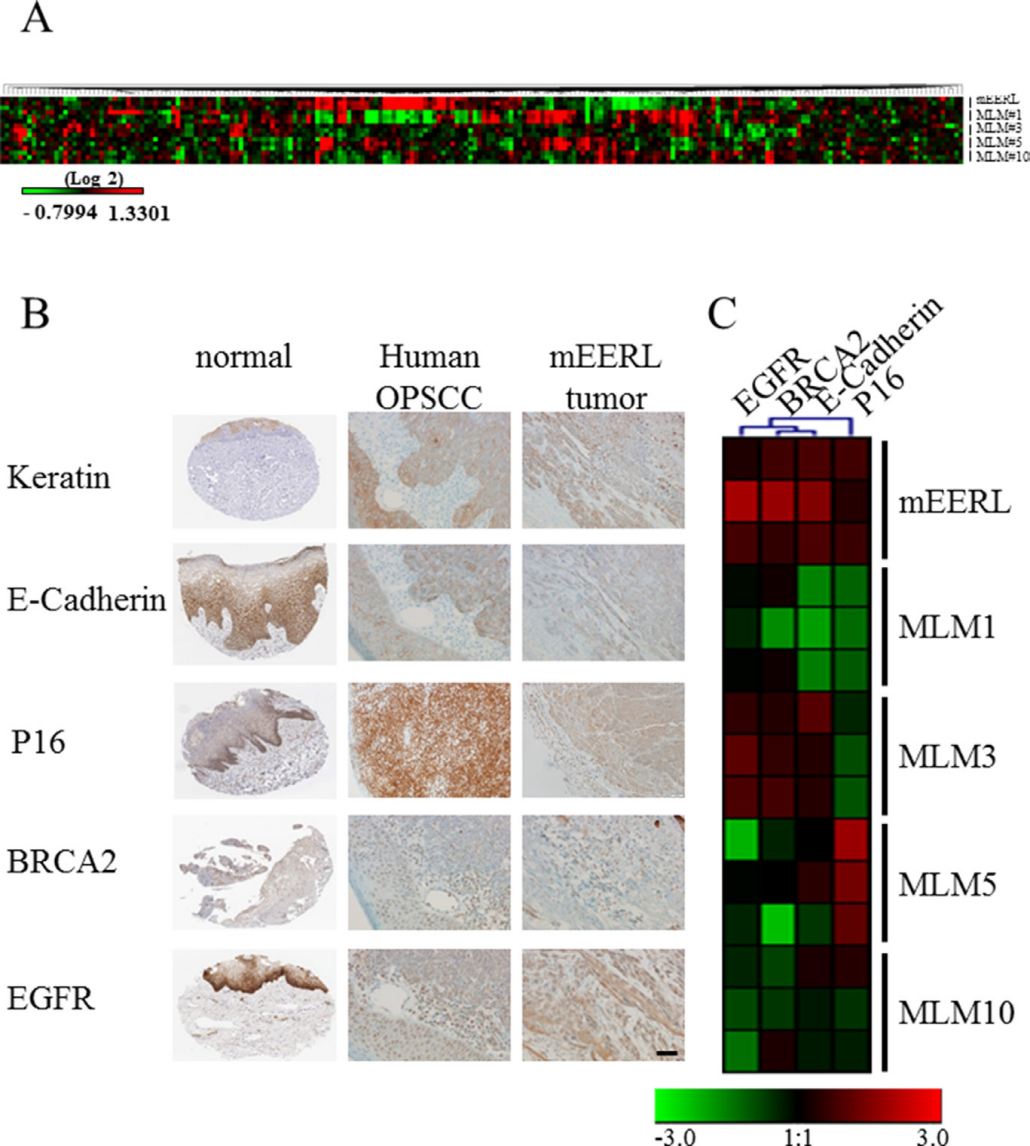
Protein expression in mEERL, MLMs and human HPV+ OPSCC (**A**) Heat map of reverse phase protein array analysis of parental mEERL cells and MLM clones performed in triplicate. (**B**) Immunohistochemical staining of normal tissue (The Human Protein Atlas) (38), human HPV+ OPSCC and mEERL tumor for hallmark proteins of OPSCC (Keratin, E-cadherin, P16, BRCA2 and EGFR). Scale bar, 40 μm. (**C**) RPPA heatmap of protein expression of four markers (EGFR, BRCA2, E-cadherin and P16) analyzed in panel B. Cytokeratin was not analyzed as it was not included in the RPPA.

Reverse phase protein array (RPPA) analysis further validated the heterotypic nature of the MLM cell lines, (Figure 3A) demonstrating differences in their protein expression. In many instances MLM protein expression differs significantly from the parental line; most strikingly for MLM#1. Taken together, these data are consistent with the published literature emphasizing the heterogeneity of metastasis.

### mEERL and MLM tumors mimic human HNSCC tumor staining profiles

Human metastatic OPSCC samples are rarely biopsied, making a direct molecular comparison with the MLM clones difficult. While tissue microarrays containing limited numbers of metastatic samples are commercially available (US Biomax, Inc), the HPV status of these samples is unknown limiting their utility for our system. Thus, we validated the expression of epithelial and tumor markers characteristic of human HPV+ OPSCC with the mEERL model of primary disease. HPV tumor status was confirmed by PCR for HPV16 E6 (Supplementary Figure 3). Human HPV+ OPSCCs up-regulate the cell cycle protein P16 and DNA repair protein BRCA2, yet demonstrate low expression of tyrosine kinase receptors such as EGFR while maintaining epithelial markers including cytokeratin and E-cadherin [19]. Immunohistochemical staining shows that similar to human HPV+ OPSCCs, mEERL tumors retain expression of epithelial markers (cytokeratin and E-cadherin), as well as BRCA2 and EGFR while demonstrating increased expression of P16 (Figure 3B). These data were correlated with the RPPA expression analysis of mEERL and MLM clones and further demonstrate that not only are the MLM clones heterogenous from their parental mEERL cells but also from each other (Figure 3C).

### Cellular physiology of the MLM clones and parental mEERL tumor cells

**Figure 4:**
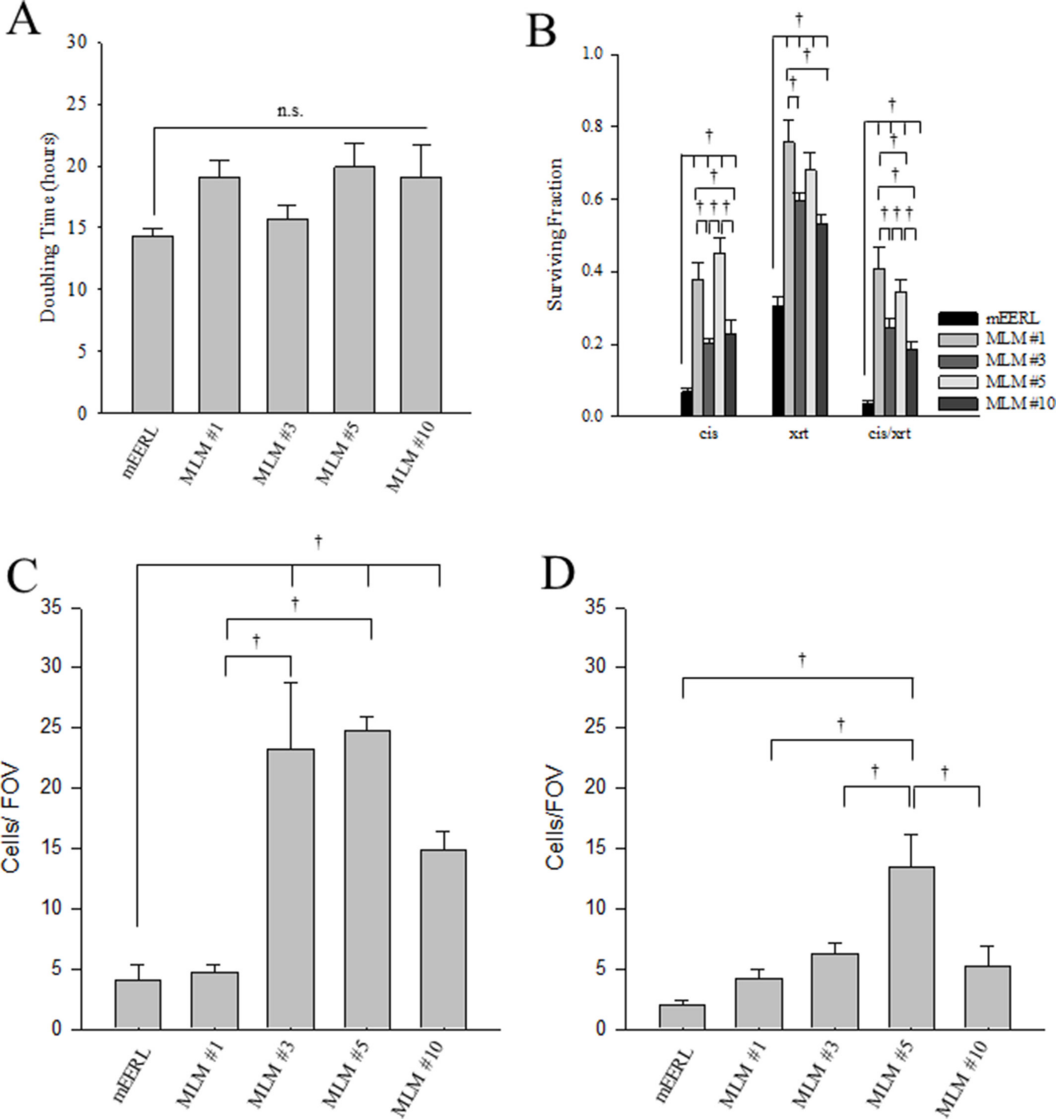
*In vitro* cellular physiology in the MLM clones (**A**) *In vitro* growth rate of the parental mEERL cells compared to the MLM clones. Growth is shown as doubling time (calculated as DT = Tln(2)/ln(xE/xb) where DT is doubling time; T is the time period; and xE or xB is the number of cells at the ending or beginning of the time period), each bar represents the mean ± SEM, *N* = 12 from three independent experiments. Differences in growth between the clones did not show statistically significant differences based on ANOVA (ns., *P* = 0.192). (**B**) Clonogenic survival of parental mEERL cells compared to the MLM clones. Cells were treated with 2 μM cisplatin, 4 Gy x-ray radiation or the combination of the two modalities. Experiments were repeated three times with similar results. Each bar represents an *N* = 8 from two independent experiments; values, means ± SEM. Statistically significant differences at day 6 after treatment, based on ANOVA: ^†^*P* ≤ 0.01. (**C**) Bar graph showing cell migration and (**D**) invasion on Matrigel chambers. Bars represent an *N* = 7, experiments were repeated 3 times with similar results; values, means ± SEM. Statistically significant differences 12 hours after seeding, based on ANOVA: ^†^*P* ≤ 0.01.

To assess the physiology of the metastatic cell lines, cellular *in vitro* growth rate was analyzed. Cells were seeded at sub-confluent levels and cell number followed over time. None of the MLM clones demonstrated statistically different growth from the parental cells, but they show a trend towards slower *in vitro* growth as demonstrated by the doubling time (Figure 4A and Supplementary Figure 4A). These data suggest that no major changes in growth rate exist between the parental mEERL cells and the MLM clones *in vitro*.

Since the MLM clones were harvested from a mouse with primary tumor recurrence following standard cisplatin radiation therapy (CRT), we tested whether the MLM clones differ in sensitivity to these treatments using an *in vitro* clonogenic assay. Interestingly, each MLM cell line was significantly more resistant than the parental mEERL line to the effects of cisplatin, radiation and their combination (cisplatin *p* ≤ 0.01, radiation *p* ≤ 0.001, and cisplatin/radiation *p* ≤ 0.001) (Figure 4B and Supplementary Figure 4B). Additionally, there are significant differences between the clones. For cisplatin treatment, MLM#1 and #5 are more resistant than MLM#3 and #10 (*p* ≤ 0.01); for radiation, MLM#1 is more resistant than MLM #3 or MLM#10 (*p* ≤ 0.01) with none of the other clones showing significant differences between each other. When cisplatin and radiation treatment modalities are combined, MLM#1 and #5 are significantly more resistant than MLM#3 and #10 (*p* ≤ 0.01). In addition, MLM#1 and MLM#5 differ significantly from each other with combined therapy (*p* = 0.001). These findings suggest that treatment resistance accounts, at least in part, for metastatic survival of these clones.

Invasion is often viewed as a requisite event to metastasis [20]. Thus, we assessed the migratory and invasive potential of the MLM clones using matrigel chambers. In the migration assay MLM#3, MLM#5, and MLM#10 show a significantly increased migratory capacity compared to the parental mEERL cells (Figure 4C and Supplementary Figure 4C) (*p* ≤ 0.01). Additionally, MLM#3 and MLM#5 are more migratory than MLM#1 and #10 (*p* ≤ 0.001). While all of the MLM clones show slight increases in invasion, only MLM#5 was significantly different from the parental mEERL cells (*p* ≤ 0.01) (Figure 4D and Supplementary Figure 4D). The lack of invasive differences between clones combined with the overall low number of cells showing migratory capacity prompted us to investigate the metastatic potential of the MLM clones *in vivo*.

### MLM clones are capable of *in vivo* growth and secondary metastasis

**Figure 5:**
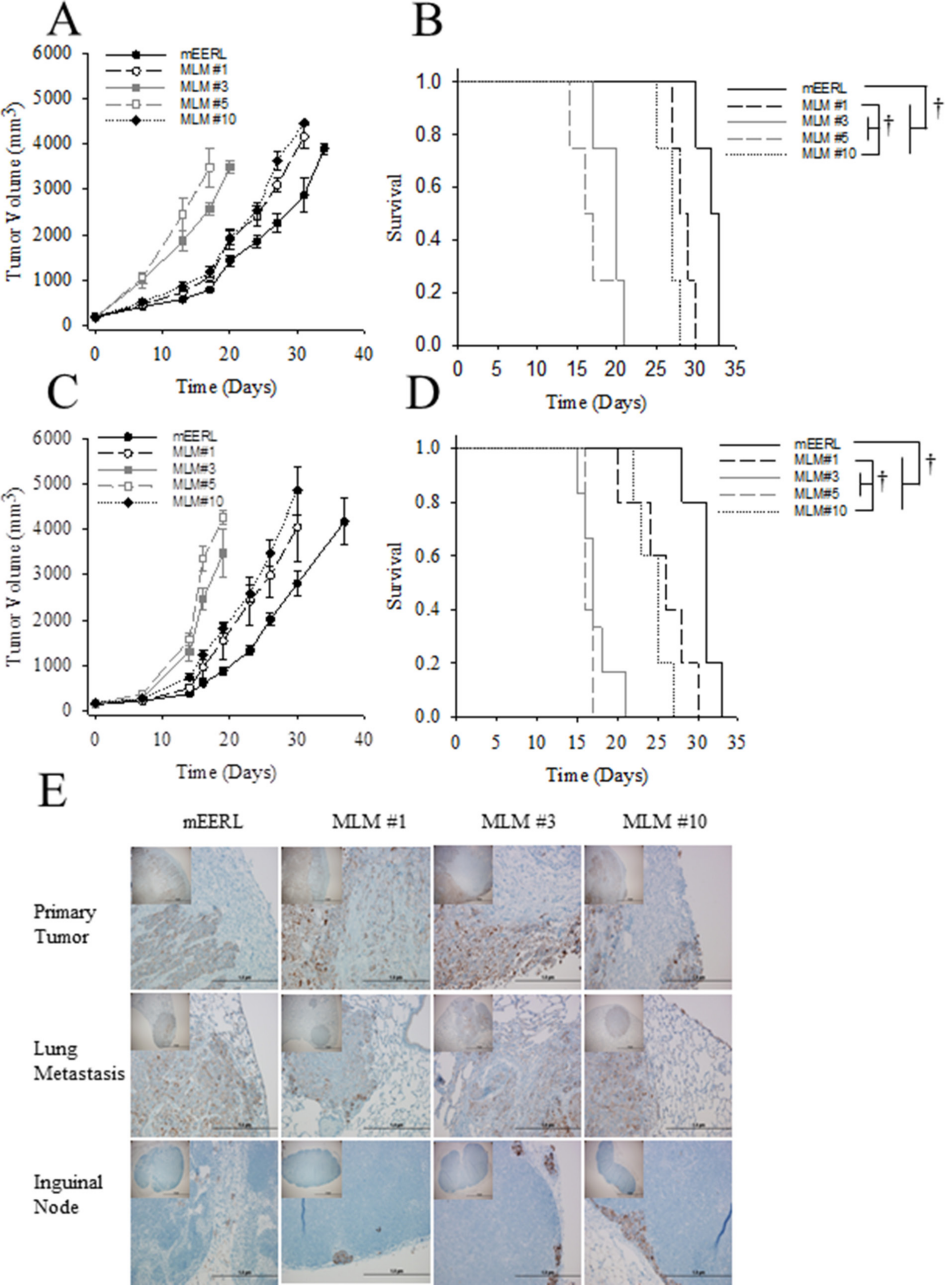
*In vivo* growth of MLM clones Parental mEERL cells and MLM clones were injected into the hind limb of mice (50,000 cells/mouse *N* = 5 mice/group) to assess MLM growth *in vivo*. (**A**) Tumor growth and (**B**) mouse survival in wild type C57Bl6 mice. (**C**) Tumor growth and (**D**) mouse survival in immune incompetent Rag1 mice. Values (A, C) mean, ± SEM. Kaplan Meier survival plot differences (B, D) were calculated by pairwise multiple comparison procedures (Holm-Sidak method): ^†^*P* ≤ 0.01. (**E**) Immunohistochemical analysis of tissues harvested from the C57Bl/6 mice, (experiment in panel A). Pan-cytokeratin staining (brown) indicates epithelial cells in each section. 4×, inset, and 40× magnification. Scale bars represent 100 μm and 1 μm respectively.

To begin characterizing the MLM model system *in vivo*, 5 × 10^4^ mEERL or MLM cells were implanted in C57Bl/6 mice. Each mouse developed tumor and tumor growth was followed until sacrifice criteria were met. In contrast to our *in vitro* results (Figure 4A) MLM clones #3 and #5 grew at a vastly increased rate compared to MLM #1, #10 or mEERL while MLM#1 and #10 only slightly outgrew the parental line (Figure 5A). Consequently, the increased tumor growth rate resulted in a statistically shorter survival for MLM#3 and MLM#5 (*p* ≤ 0.007) (Figure 5B).

Given the role of the immune system in recognizing and clearing tumors in this HPV+ model of OPSCC [21], we wondered if the difference in growth rates was due in part to failed immune recognition of these MLM tumors. Therefore, the study was repeated in C57Bl6/Rag1 mice lacking functional T and B cells. Interestingly, tumor growth and survival patterns did not differ from those in wildtype C57Bl/6 mice (Figure 5C, 5D). Although we cannot rule out a role of the innate immune response, these data suggest that the increased *in vivo* growth rate observed in MLM#3 and #5 is not related to evasion of an adaptive immune response.

To assess the metastatic potential of the MLM clones, tissue was harvested from these mice. Mice exhibiting gross pulmonary metastasis had the primary tumor, draining (inguinal) lymph node, and lungs sectioned and stained for cytokeratin to visualize epithelial cells. While many animals showed no gross evidence of metastasis (notably no mice in MLM#5), the limited experimental duration (due to rapid primary tumor growth) potentially obscured identification of metastatic outgrowth. Of those mice that did have macroscopic lung metastasis, positive nodules of cytokeratin staining in the inguinal lymph nodes (Figure 5E) were also present suggesting the MLM lines spread via a lymphatic route similar to the human disease [22]. Together, the data demonstrate that MLM cells are not only capable of *in vivo* growth but also metastasis.

### *In vivo* response to standard cisplatin/radiation therapy differs between the MLM clones

**Figure 6:**
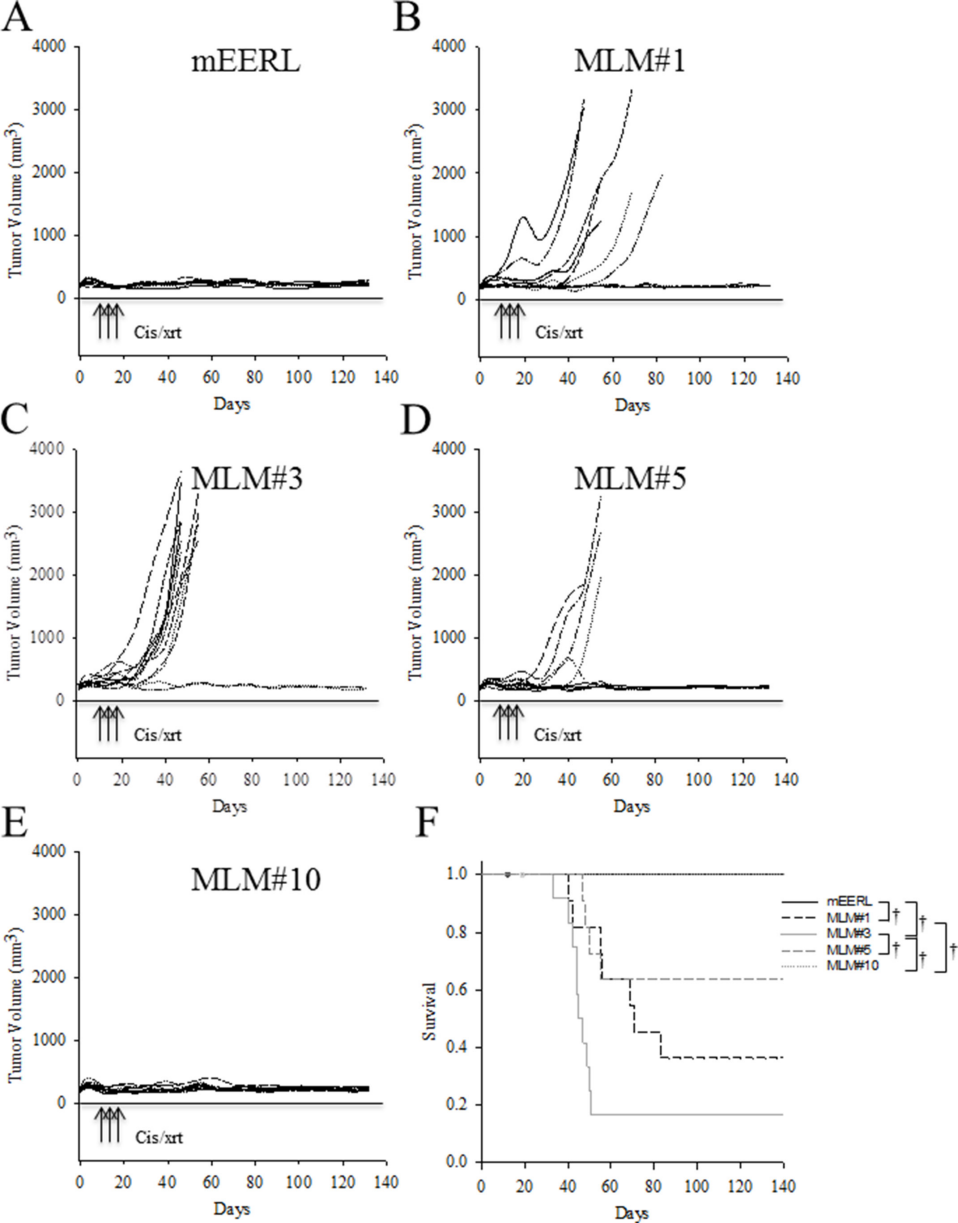
*In vivo* response to standard cisplatin/radiation therapy Parental mEERL cells and MLM clones were injected into the hind limb of C57Bl6 mice (50,000 cells/mouse *N* = 12 mice/group). After establishment of palpable tumors, mice were treated with IP cisplatin (20 mg/m2) and x-ray radiation (8 Gy) on days 4, 11, and 18. Individual mouse tumor growth curves for each cell line, panel (**A**) mEERL, (**B**) MLM#1, (**C**) MLM#3, (**D**) MLM#5, and (**E**) MLM#10. (**F**) Kaplan Meier tumor free survival graph. All non-surviving mice were sacrificed due to tumor burden at the primary (hind limb) tumor sight. Deaths not associated with tumor (death during CRT) were censored from the data and are indicated by dots on the corresponding curve. Statistically significant differences were calculated by pairwise multiple comparison procedures (Holm-Sidak method): ^†^*P* ≤ 0.01.

Because the *in vivo* growth rate of the clones differed from that observed *in vitro* (Figures 4A and 5A), we asked whether the treatment resistance inherent to the MLM cell lines *in vitro* would be retained *in vivo*. Briefly, 5 × 10^4^ tumor cells (parental mEERL or the MLM lines) were implanted in C57Bl/6 mice. Due to the accelerated growth rate of the MLM clones, mice were treated with standard CRT therapy early in the disease course, on days 4, 11, and 18. Although each mouse initially developed a palpable tumor, as can be seen in Figure 6A–6E, all mice in the parental mEERL (Figure 6A) and MLM #10 (Figure 6E) groups cleared their primary disease. Of the remaining three clones, MLM#3 (Figure 6C) was the most resistant to treatment with only a 20% survival rate, while MLM#1 (Figure 6B) and #5 (Figure 6D) demonstrated 50% and 67% survival respectively (Figure 6F). It is notable that although the *in vitro* resistance to cis/xrt is similar between MLM#3 and MLM#10 (Figure 4B), the survival response *in vivo* differs significantly (*p* < 0.001, Figure 6F). These data not only demonstrate that the MLM clones differ in therapeutic resistance from the parental mEERL cells, but also exemplify the clonal heterotypic differences in treatment response. As in the growth rates, the resistance demonstrated *in vivo* is not completely reflective of those shown *in vitro*.

### MLM clones have an increased rate of metastasis when re-implanted in mice

**Figure 7:**
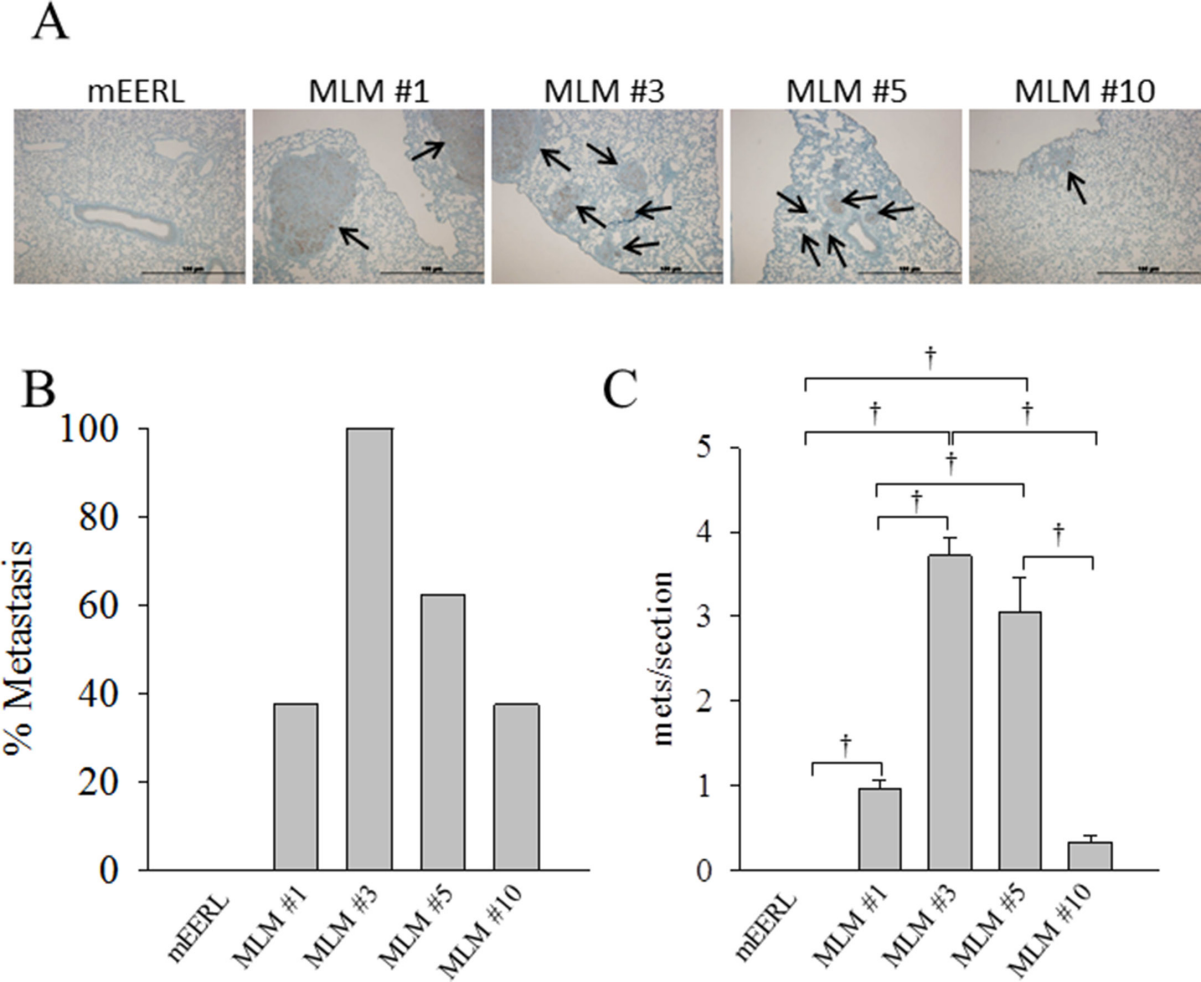
Metastatic potential of MLM clones Parental mEERL cells and MLM clones were injected into the hind limb of C57Bl6 mice (50,000 cells/mouse *N* = 8 mice/group) and allowed to establish tumor for 4 days. The tumor was treated with 8 Gy x-ray radiation on days 7, 9, and 11 to slow growth of the primary tumor and increase time for metastasis to develop. At day 35 all mice were sacrificed. (**A**) Representative IHC image of keratin positive lung metastasis from each clone 4× magnification. Scale bars represent 100 μm. (**B**) Percent of mice from each clone demonstrating lung metastasis. (**C**) Number of keratin positive metastases per section (7–8 mice per group 3 independent sections/mouse); values, mean, ± SEM. Statistically significant differences at day 35 after tumor implantation, based on ANOVA: ^†^*P* ≤ 0.01.

To define the metastatic rate of MLM cells, we analyzed their spread to distant organs at equivalent end- points. The accelerated growth rate of the MLM lines *in vivo* necessitated that primary tumor sites be irradiated in an attempt to prolong survival, thus allowing sufficient time for metastatic outgrowth. Briefly, after establishment of a palpable primary tumor, each mouse was treated with 8 Gy radiation on days 4, 6, and 8 after tumor implantation. Tumor growth was monitored weekly until sacrifice criteria were met in the first mouse (day 35); at this time, tissues from all mice were collected. Pulmonary metastasis was assessed by cytokeratin staining, representative images are shown in Figure 7A. Under these conditions, parental mEERL cells failed to metastasize while all the MLM clones showed some level of pulmonary spread with MLM#3 metastasizing in every animal (Figure 7B). Although not statistically different from each other, MLM#3 and MLM#5 demonstrate a significant increase in number of metastases compared to clones #1 and #10 (*p* ≤ 0.029) (Figure 7C). Not only did MLM#3 show the highest rate of metastasis but also the highest total number of metastatic tumors. These data reflect the MLM clones increased resistance to radiation (Figure 4B), but also reveal differences in metastatic rate between lines with similar radiation resistance (MLM#3 and MLM#10). Thus, in addition to their heterogeneous growth and treatment response, the metastatic clones differ in their ability to metastasize to the lung.

## DISCUSSION

Clinically relevant animal models must recapitulate critical aspects of the human disease. Here, we characterize a novel mouse model of recurrent/metastatic HPV+ OPSCC that faithfully mimics key aspects of: 1) heterogeneity, 2) anatomically relevant metastasis, and 3) resistance to standard first-line CRT. While all MLM cell lines were harvested from the lungs of a single mouse injected with clonal parental mEERL cells, we show that each metastatic line derived from this animal is phenotypically distinct from the parental line and also from each other. These differences exist not only in gene and protein expression profiles, but also in growth rates, resistance to CRT and metastatic potential (Figures 2–7). Such heterogeneity is consistent with the published literature [23–25]. Interestingly, many of the *in vitro* data were not reflective of *in vivo* physiology. This is likely due to the influence of factors present in the tumor microenvironment (stromal and immune cells) that are absent *in vitro*. These differences further emphasize the need for *in vivo* model systems.

On the surface, heterogeneity of solid tumors and their metastases presents a bleak outlook for cancer cures. However, it is important to note that in this system, all MLM cell lines retained the cellular manifestations of HPV oncoprotein (E6 and E7) expression strongly suggesting that these viral oncogenes drive key pathways necessary for tumor cell survival (Figure 1C and 1D). These data suggest that while metastatic heterogeneity exists, it is likely that common pathways for growth and survival are utilized. Once identified, these pathways could be therapeutically targeted to control or eliminate metastatic growth despite heterogeneity.

Local spread to the draining lymph nodes and distant pulmonary metastasis (Figure 5E and Figure 7A) in the MLM model system mirror the clinical progression for human OPSCC [26–27]. While the percent lung metastasis varied among the MLM lines, the finding that they all honed to the lung suggests a shared ability to target this organ. Further genotype and expression analyses will help define the pathways targeting the lung and sustaining tumor growth at this site.

Finally, this recurrent-metastatic mouse model demonstrates resistance to standard CRT which is common in OPSCC patients that suffer disease recurrence and progression [28–29]. Unfortunately, recurrence poses a major hurdle for these patients as currently the cancer therapeutic arsenal offers little to successfully combat this complication. This metastatic mouse model offers the ability to test the efficacy of drugs emerging from the therapeutic pipeline in blocking metastasis and recurrence due to resistance. It is important to note that this model is immune competent. This is of particular importance to HPV+OPSCCs as the immune system plays a significant role in tumor clearance [30]. Thus, this model provides a system in which to test new drugs and study their interplay with the immune system in controlling or eliminating metastatic tumor growth.

The parental mEERL cell line exemplifies many of the original “hallmarks of cancer” described by Hanahan and Wienberg over a decade ago [31]. For example, the mEERL model system possesses replicative immortality [21], sustained proliferative signaling [32–33], resistance to cell death (unpublished data), and evasion of growth suppressors [34]. Moreover, two emerging hallmarks of tumor cells [8] are also exemplified in this model system: 1) dysregulation of cellular energetics [35] and 2) immune evasion [36]. In this report, we describe the mEERL model's ability to replicate the remaining cancer hallmarks: invasion and metastasis. Together, the mEERL and MLM models provide a system with which to study the mechanisms driving tumor growth and metastasis as well as providing paradigms for testing new therapies aimed at blocking tumor progression and recurrence. The metastatic OPSCC model in particular holds great promise for clinical advancement in this field as currently patients with recurring tumors have limited options. This model not only provides an immune competent *in vivo* system for drug testing but also offers a system in which to molecularly define metastatic pathways and identify novel targets for therapeutic intervention.

## MATERIALS AND METHODS

### Cell lines, culture conditions and authentication

Primary mouse oropharyngeal epithelial cells (MOE) from male C57Bl/6 mice were isolated and cultured in KSFM medium (Gibco Life Technologies). Stable MOE lines expressing HPV16 E6/E7, hRas, and luciferase (mEERL) were previously generated and maintained in E-media [37, 16, 15]. mEERL lung metastasis cells (MLM) were isolated from the lungs of a treatment failed mouse. Individual lung metastasis were dissected, homogenized and dissociated with 2 U/mL dispase (Roche) in RPMI1640 with penicillin/streptomycin and Fungizone (Gibco Life Technologies). The resulting cells were washed in PBS resuspended in E-media with Fungizone and seeded on 35 mm tissue culture dishes. Each metastatic clone was subsequently expanded to larger vessels, used for analysis and cryopreserved. In this paper, we authenticate the MLM cell lines as being derived from the parental mEERL tumor (Figure 1). As a further indication that the MLM cell lines are murine in origin, we are able to implant them into immune competent syngeneic mice and observe tumor growth (Figure 5).

### Western blot

Cells were grown to 80% confluence and harvested in lysis buffer (50 mM Tris HCl pH 7.5, 150 mM NaCl, 5 mM EDTA, 2 mN Na_3_VO_4_, 100 mM NaF, 10 mM NaPPi, 10% glycerol, 1% Triton X-100, 17.4 μg/mL paramethylsulfonylfluoride, 1X HALT with EDTA), 1% Tx-100 and HALT with EDTA (Peirce). Lysates were spun at 10,000 RPM for 15 minutes at 4°C. Tx100 soluble cell lysates (40 μg /lane) were separated by SDS PAGE and analyzed by western blot with the following antibodies: PTPBl (scH300 Santa Cruz Biotechnology), P53 (1C12 Cell Signaling), pRb (sc7905 Santa Cruz Biotechnology), and βactin (AC-74 Sigma). Standard HRP secondary antibodies (1:10,000) and ECL reagent (Thermo) were used for visualization with a CCD camera imaging system (UVP).

### Luciferase expression

Luciferase expression assays were conducted on the soluble fraction of cell lysates harvested as per the manufacturer's instructions (E1500 Promega). Luciferase was measured by incubating 10 μg protein lysate with 50 μl substrate and read on a Promega GLOMAX 96 microplate luminometer.

### PCR and Illumina microarray analysis

RNA was harvested as follows: Cells were grown to approximately 80% confluence, rinsed with 1X phosphate buffered saline and lysed in 200 μl TRIZOL Reagent (Life Technologies) as per the manufacturer's directions. RNA was purified on RNeasy Mini Column (Qiagen) and samples were eluted in RNase free deionized water. For RT-PCR, cDNA was generated using the Retroscript Kit (Life Technologies). Standard PCR was performed to assess expression of HPV16 E6, E7, Ras and GAPDH in each sample with the indicated primers:

HPV16 E6 forward primer 5′CAAACCGTTGTGT GATTTGTTAATTA 3′

HPV16 E6 reverse primer 5′GCTTTTTGTCCAGA TGTCTTTGC 3′;

HPV16 E7 forward primer 5′ ATGCATGGAGATA CACCTACATTGCATG 3′

HPV16 E7 reverse primer 5′ TTATGGTTTCTGA GAACAGATGGGGC 3′

hRas forward primer 5′ ATGACGGAATATAAGC TGGTGGTGG 3′

hRas reverse primer 5′ CATGGCGCTGTACTC CTCCTG 3′

GAPDH forward 5′ GGGAAGGTGAAGGTCGG AGT-3′

GAPDH reverse 5′ TGGAAGATGGTGATGGG ATTTC-3′.

RNA samples were also subjected to gene expression profiling using Illumina MouseRef-8 Expression BeadChips (Illumina). Raw expression data were subjected to cubic spline normalization in GenomeStudio (version 2011.1). Principal component analysis (PCA) were performed with Partek Genomics Suite (version 6.6) using a significance of *p* < 0.01 as a threshold for gene inclusion. ANOVA and *T*-test were performed using in-house R scripts, and the significant genes were obtained using a False Discover Rate (FDR: Benjamini–Hochberg–Yekutieli procedure) of *p* < 0.05. Hierarchical clustering was performed using the software GENESIS (version 1.7.6). These data have been deposited in GEO (accession # GSE68935).

### Reverse phase protein microarray (RPPA)

Briefly, lysates from mEERL and MLM clones were harvested from 35 mm dishes grown to 80% confluence and shipped to: MD Anderson Core Facility – Functional Proteomics – RPPA (http://www.mdanderson.org/education-and-research/resources-for-professionals/scientific-resources/core-facilities-and-services/functional-proteomics-rppa-core/index.html) for analysis. Heatmap was generated using protein expression profiles across samples. Proteins and samples were clustered using an average linkage hierarchical clustering algorithm.

### Cell proliferation assay

Cellular growth rate was determined by seeding 5000 cells per well in 12 well plates. Time points were collected in triplicate by washing in PBS–/– with 2 mM EDTA and harvesting with TrypLE (Life Technologies) at 24, 48, 72, and 96 hours. All replicates were counted on a Cell Countess system (Invitrogen). Doubling times were calculated as DT = Tln(2)/ln(xE/xB) where DT is doubling time; T is the time period; and xE or xB is the number of cells at the ending or beginning of the time period, respectively.

### Colony formation

Colony forming assays were conducted by plating 200 cells per well in 12 well dishes. Six hours after seeding 1000X cisplatin (Calbiochem) solubilized in DMSO was added to a final concentration of 2 μM. Control plates and plates receiving radiation alone were treated with an equivalent volume of DMSO. Within 10 minutes of cisplatin addition, 4 Gy radiation was administered to the radiation alone or the cisplatin/radiation conditions. Plates were returned to a 37° incubator where colonies were allowed to grow for 6 days and subsequently fixed in 70% ethanol and stained with trypan blue in 10% ethanol. Colonies in triplicate wells were counted on a GelCount imaging system (OXFORD OPTRONIX) using identical settings.

### Migration and invasion

Migration and invasion assays were performed by seeding 50,000 cells per well in BD BioCoat matrigel chambers (BD Biosciences). After 20 hours, invading or migrating cells were fixed and stained as per the manufacturer's instructions and counted at 20X magnification (EVOS cell imaging system, Life Technologies).

### Animal studies

All animal experiments were approved by the Sanford Research IACUC and performed within institutional guidelines. Four to six week old, 20–25 gm male C57BlJ/6 mice or C57BlJ/6 Rag 1 mice (The Jackson Laboratory) were maintained at the Sanford Research Laboratory Animal Research Facility in accordance with USDA guidelines. Tumors were initiated as follows: using a 23-gauge needle mEERL or MLM cells were implanted subcutaneously in the right hind flank of mice (*n* = 10/group). For the indicated schedule, mice were anesthetized with 87.5 mg/Kg ketamine and 12.5 mg/Kg xylazine, and treated with 8 Gy X-ray radiation (RS2000 irradiator, RadSource Technologies, Inc. Suwanee, GA), intraperitoneal cisplatin (CalBiochem) dissolved in bacteriostatic 0.9% sodium chloride (Hospira Inc.) at 20 mg/m^2^ or the combination of both modalities. Tumor growth was measured weekly as previously described [15]. Animals were euthanized when tumor size was greater than 1.5 cm in any dimension. Conversely, mice were considered tumor free when no measurable tumor was detected for a period of two months. Survival graphs were calculated by standardizing for a tumor volume of 2500 mm^3^. Statistical analysis for the survival graphs was performed using the log-rank test with α = 0.01.

### Human subjects

All human tissues were collected with informed consent and approval from Sanford Health IRB (#MOD00000135).

### Microscopy

Paraffin embedded tissue blocks were prepared, sectioned, and stained using standard immunohistochemical techniques by the Sanford Molecular Pathology Core. Briefly, paraffin embedded tissues were sectioned to 5 μm and stained on a BenchMark^®^ XT automated slide stainer (Ventana Medical Systems, Inc). Ventana iView DAB detection kit and counterstaining with hematoxylin were used for visualization. Staining for pan-cytokeratin (ab9377, Abcam), E-cadherin (#3195, Cell Signaling), P16 (MA5-17093, Thermo and #10883-1-AP, ProteinTech), BRCA2 (HPA026815, Sigma), and EGFR (ab2430, Abcam) were used to visualize tumors of epithelial origin; exclusion of primary antibody served as the negative control. Lung metastases from three independent sections were manually counted on an (Olympus DP71) microscope.

### Statistical analysis

For statistical analysis of mircroarray data see PCR and Illumina microarray analysis methods. All other statistical analyses were performed using Sigma Plot 11 (Systat Software, Inc.). Survival plots were analyzed using Kaplan-Meier survival curves and statistical significance was determined by the log-rank test, multiple comparisons were made with the Holm-Sidak method. For all other data one way ANOVA with the Holm-Sidak pairwise multiple comparison procedures were used. An alpha 0.05 was used for all tests unless otherwise indicated.

## SUPPLEMENTARY MATERIALS FIGURES AND TABLES


