# Supplementary material for: Metastatic model of HPV+ oropharyngeal squamous cell carcinoma demonstrates heterogeneity in tumor metastasis
Source: Oncotarget. 2016 Mar 22;7(17):24194–207. doi: 10.18632/oncotarget.8254 (PMC5029694; doi:10.18632/oncotarget.8254)
Supplement: Supplementary file 1 [file oncotarget-07-24194-s001.pdf]

Metastatic model of HPV+ oropharyngeal squamous cell carcinoma demonstrates heterogeneity in tumor metastasis

Supplementary Materials

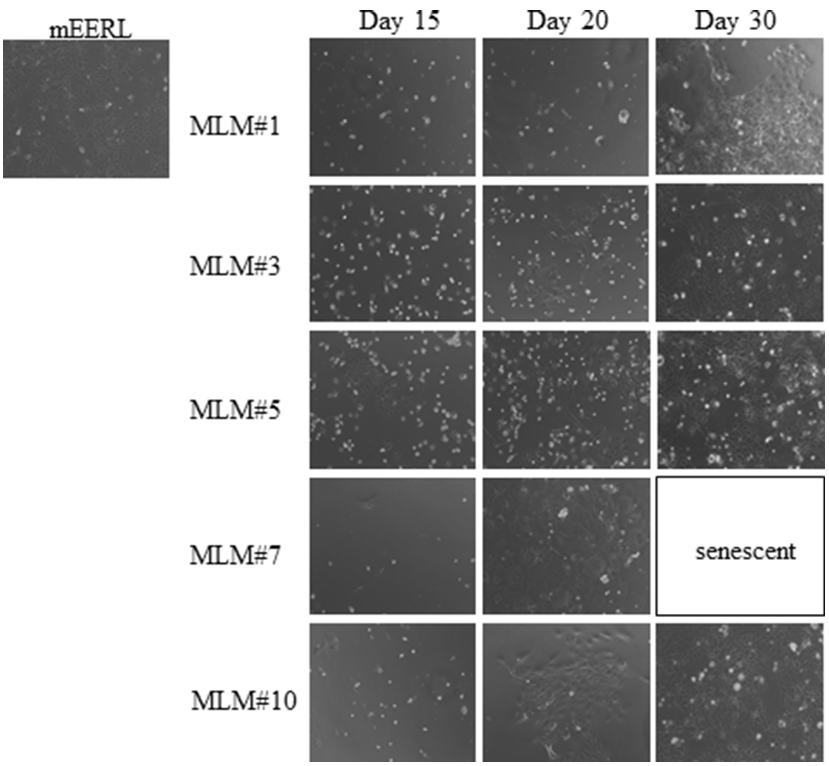

Supplementary Figure S1: Photomicrograph of cultured cells from individual lung tumors compared to parental tumor cells at the indicated day.

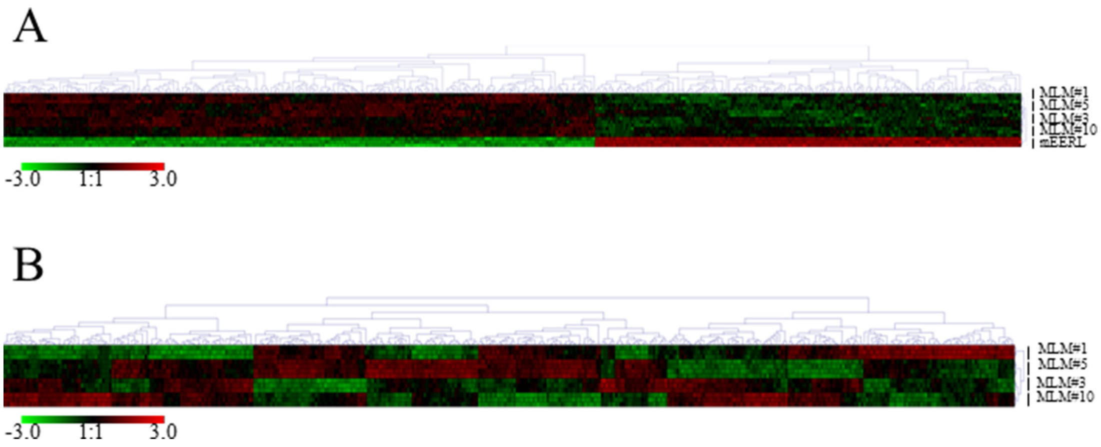

Supplementary Figure S2: Hierarchical clustering analysis was performed for two sets of DEGs (Differentially Expressed Genes). Both genes and samples are clustered using an average linkage clustering algorithm. (**A**) Clustering analysis of DEGs in a comparison between parental mEERL cells and MLM clones. DEGs were determined based on *T*-test with a False Discover Rate (FDR: Benjamini–Hochberg–Yekutieli procedure) of $p < 0.05$. (**B**) Clustering analysis of DEGs among MLM clones. DEGs were determined based on ANOVA with a False Discover Rate (FDR: Benjamini–Hochberg–Yekutieli procedure) of $p < 0.05$. Due to instrument error, one of the replicates for MLM5 was run two times.

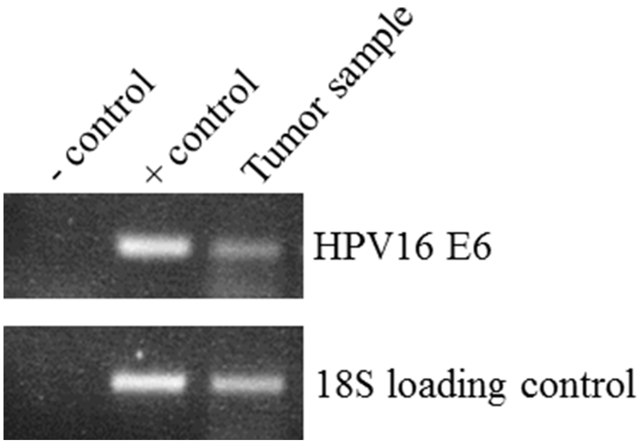

Supplementary Figure S3: PCR amplification of HPV16 E6 of DNA isolated from an OPSCC patient. Controls included no template (-control) and DNA from a known HPV+ OPSCC cell line (+control). 18S served as a loading control. Tumor from this OPSCC patient was utilized for IHC validation in Figure 3B.

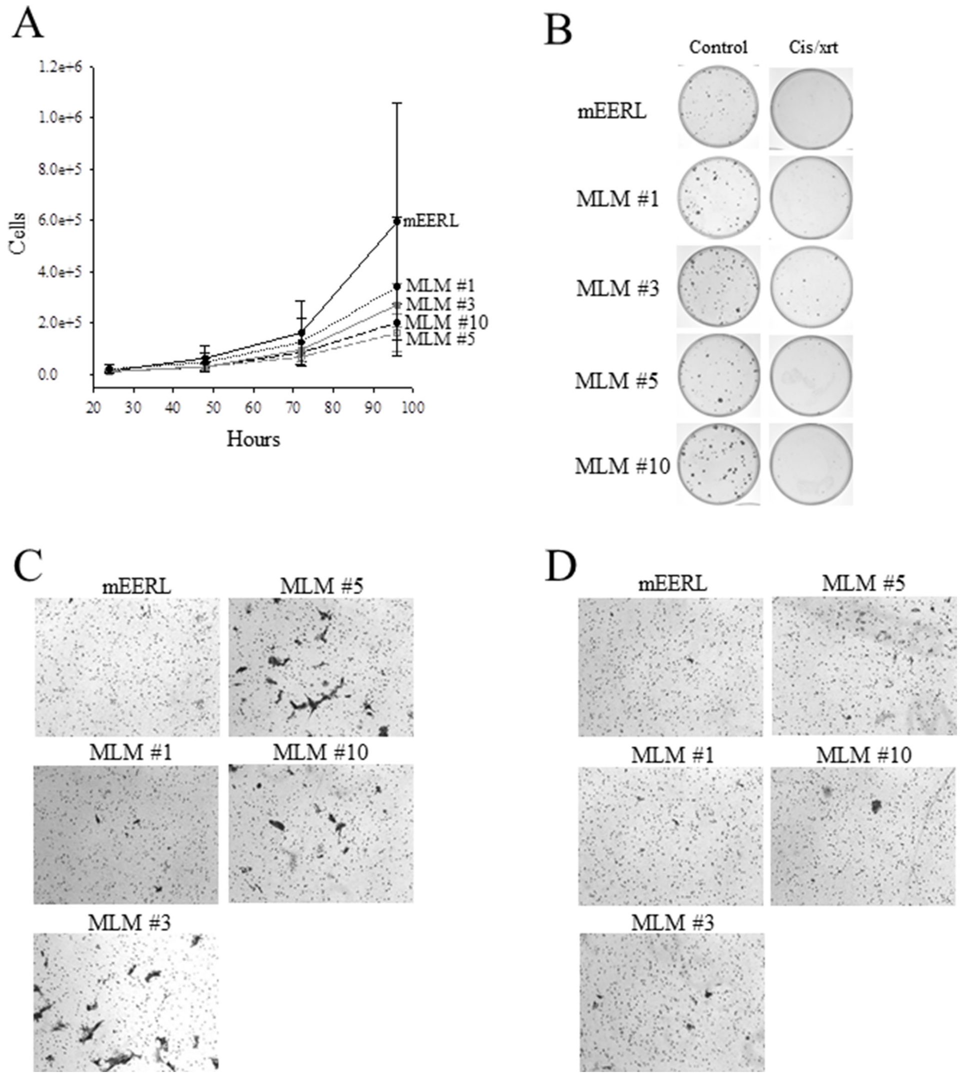

Supplementary Figure S4: (**A**) Tumor growth of mEERL and MLM lines over time. Data points are an average of $N = 12$ from three independent experiments. Data points, mean ± SEM. (**B**) Representative image of a clonogenic assay showing the effects of cisplatin, radiation, or the combined modality on clonogenic survival. (**C**) Representative picture of migration and (**D**) invasion through traditional matrigel inserts.

Supplementary Table S1: Pathway analysis of DEGs: mEERL and MLMs

Ingenuity Canonical Pathways	-log (*p*-value)
IL-8 Signaling	6.60E + 00
ATM Signaling	6.54E + 00
CXCR4 Signaling	6.26E + 00
STAT3 Pathway	5.65E + 00
Inhibition of Angiogenesis by TSPl	5.33E + 00
HMGB1 Signalling	5.19E + 00
Molecular Mechanisms of Cancer	5.17E + 00
IL-22 Signaling	5.16E + 00
Thrombin Signaling	5.02E + 00
GDNF Family Ligand-Receptor Interactions	5.02E + 00
Pancreatic Adenocarcinoma Signaling	4.96E + 00
Glioma Invasiveness Signaling	4.71E + 00
UVC-lnduced MAPK Signaling	4.69E + 00
N-acetylglucosamine Degradation II	4.48E + 00
Cardiac Hypertrophy Signaling	4.23E + 00
Leukocyte Extravasation Signaling	4.22E + 00
Role of NFAT in Cardiac Hypertrophy	4.08E + 00
L-17 Signaling	3.97E + 00
Ephrin B Signaling	3.92E + 00
IL-17A Signaling in Gastric Cells	3.88E + 00

Top twenty significantly enriched pathways. Enriched pathways in the first set of DEGs obtained from a comparison between parental mEERL cells and MLM clones.

Supplementary Table S2: Pathway analysis of DEGs: among MLMs

Ingenuity Canonical Pathways	-log (*p*-value)
Aryl Hydrocarbon Receptor Signaling	3.96E + 00
Signaling by Rho Family GT Pases	3.35E + 00
Role of NANOG in Mammalian Embryonic Stem Cell Pluripotency	3.30E + 00
Glioblastoma Multiforme Signaling	3.15E + 00
Ascorbate Recycling (Cytosolic)	3.10E + 00
LI-8 Signaling	3.01E + 00
Molecular Mechanisms of Cancer	2.95E + 00
Spermine and Spermidine Degradation 1	2.8GE + 00
Hepatic Fibrosis / Hepatic Stellate Cell Activation	2.78E + 00
Semaphorin Signaling in Neurons	2.76E + 00
Role of Tissue Factor in Cancer	2.64E + 00
Axonal Guidance Signaling	2.64E + 00
NRF2-mediated Oxidative Stress Response	2.52E + 00
CCR3 Signaling in Eosinophils	2.49E + 00
ILK Signaling	2.43E + 00
PC pathway	2.43E + 00
Role of Macrophages, Fibroblasts and Endothelial Cells in Rheumatoid Arthritis	2.40E + 00
Thrombin Signaling	2.35E + 00
Antioxidant Action of Vitamin C	2.25E + 00
Nitric Oxide Signaling in the Cardiovascular System	2.21E + 00

Top twenty significantly enriched pathways. Enriched pathways in the second set of DEGs obtained from a comparison among MLM clones.

Supplementary Table S3: Pathway analysis between the two sets of DEGs

Ingenuity Canonical Pathways	-log (*p*-value)
Sulfite Oxidation IV	2.95E + 00
Spermine and Spermidine Degradation 1	2.34E + 00
Melatonin Degradation II	2.34E + 00
N-acetylglucosamine Degradation II	2.34E + 00
CMP-N-acetylneuraminate Biosynthesis 1 (Eukaryotes)	2.25E + 00
Mineralocorticoid Biosynthesis	1.87E + 00
Bile Acid Biosynthesis, Neutral Pathway	1.84E + 00
Glucocorticoid Biosynthesis	1.84E + 00
Phenylalanine Degradation IV (Mammalian, via Side Chain)	1.80E + 00
Androgen Biosynthesis	1.77E + 00
Putrescine Degradation III	1.72E + 00
Tryptophan Degradation X (Mammalian, via Tryptamine)	1.69E + 00
LPS/IL-1 Mediated Inhibition of RXR Function	1.60E + 00
Estrogen-mediated S-phase Entry	1.57E + 00
Dopamine Degradation	1.57E + 00
Superpathway of Methionine Degradation	1.45E + 00
Xenobiotic Metabolism Signaling	1.43E + 00
Cell Cycle Regulation by BTG Family Proteins	1.41E + 00
Noradrenaline and Adrenaline Degradation	1.41E + 00
Inhibition of Matrix Metalloproteases	1.36E + 00

Top twenty significantly enriched pathways. Enriched pathways in shared genes between the first and the second set of DEGs.
